# Comparing the New Interdisciplinary Health in Work Intervention With Conventional Monodisciplinary Welfare Interventions at Norwegian Workplaces: Protocol for a Pragmatic Cluster Randomized Trial

**DOI:** 10.2196/36166

**Published:** 2022-04-07

**Authors:** Anje Christina Höper, Christoffer Lilja Terjesen, Nils Fleten

**Affiliations:** 1 Occupational Health in the North Department of Community Medicine UiT The Arctic University of Norway Tromsø Norway; 2 Department of Occupational and Environmental Medicine University Hospital North Norway Tromsø Norway; 3 Department of Rehabilitation University Hospital North Norway Tromsø Norway; 4 Norwegian Labour and Welfare Administration Troms and Finnmark Tromsø Norway

**Keywords:** sickness absence, work environment, work environment intervention, health related quality of life, cluster randomized trial, cost-effectiveness-analysis, cost-benefit-analysis, health in work

## Abstract

**Background:**

Musculoskeletal and mental health complaints are the dominant diagnostic categories in long-term sick leave and disability pensions in Norway. Continuing to work despite health complaints is often beneficial, and a good work environment can improve work inclusion for people affected. In 2001, the Norwegian Labour and Welfare Administration began to offer *inclusive work measures* to improve the psychosocial work environment and work inclusion of people with health complaints. In 2018, the Norwegian Labour and Welfare Administration and specialist health services started offering the new collaborative *Health in work* program. Its workplace intervention presents health and welfare information that may improve employees’ coping ability regarding common health complaints. It encourages understanding of coworkers’ health complaints and appropriate work adjustments to increase work participation.

**Objective:**

This protocol presents an ongoing, 2-arm, pragmatic cluster-randomized trial. Its aim is to compare the effect of monodisciplinary *inclusive work measures* (treatment as usual) and interdisciplinary *Health in work* in terms of changes in overall sickness absence, health care use, health-related quality of life, and costs. The secondary objectives are to compare changes in individual sickness absence, psychosocial work environment, job and life satisfaction, health, and health anxiety at both the individual and group levels.

**Methods:**

Data will be collected from national registers, trial-specific registrations, and questionnaires. Effects will be explored using difference-in-difference analysis and regression modeling. Multilevel analysis will visualize any cluster effects using intraclass correlation coefficients.

**Results:**

Inclusion was completed in July 2021 with 97 workplaces and 1383 individual consents. Data collection will be completed with the last questionnaires to be sent out in July 2023.

**Conclusions:**

This trial will contribute to filling knowledge gaps regarding the effectiveness and costs of workplace interventions, thereby benefiting health and welfare services, political decision makers, and the public and business sectors. The findings will be disseminated in reports, peer-reviewed journals, and conferences.

**Trial Registration:**

ClinicalTrials.gov NCT04000035; https://clinicaltrials.gov/ct2/show/NCT04000035

**International Registered Report Identifier (IRRID):**

DERR1-10.2196/36166

## Introduction

### Sickness Absence in Norway

Musculoskeletal and mental health complaints are the dominant diagnostic categories in long-term sickness absence and disability pensions in Norway [1-4]. Many of these complaints can be described as subjective health complaints with high prevalence in the general population [5-7]. Preventing subjective health complaints is difficult, but improving the person’s perception of the complaints as well as related coping mechanisms seems to have a positive impact in terms of sickness absence [8]. In fact, work-focused cognitive behavioral therapy and brief workplace interventions have been shown to reduce sickness absence [9-11]. Workplace interventions that presented reassuring information about low back pain based on the “non-injury-model” introduced by Aage Indahl [12] were shown to increase work participation [9,13], improve self-rated work ability, and reduce experienced complaints without changing the prevalence of low back pain [13].

For people with common health complaints, especially regarding mental health, work options often seem more beneficial than being on sick leave [14]. A Norwegian report showed that the general work environment seems to play a key role in deciding whether to go on sick leave or master one’s back pain at work [15]. Thus, workplace interventions that include both health information and workplace processes that can create a flexible and inclusive work environment seem beneficial.

To address the comparatively high rate of sickness absence in Norway [16], the government, employer-organizations, and employee-organizations signed the first Inclusive Work Agreement (Intensjonsavtale om et mer inkluderende arbeidsliv; IA-avtalen) in 2001, with the latest update in 2018 [17].

### Measures for Work Inclusion and Participation

To support workplaces in achieving the Inclusive Work Agreement goals of more inclusive work environments and reduced sickness absence, the Norwegian Labour and Welfare Administration (NAV) established NAV work centers (NWCs NAV Arbeidslivsenter) in every county [17,18]. Although NWCs offer a variety of monodisciplinary *inclusive work measure* (IWM) interventions both standardized and customized, the goal of the Inclusive Work Agreement of reducing sickness absence by 20% was not reached in the public sector [19-21], revealing a need for more effect-focused studies of IWM interventions [15].

### Background of the Health in Work Program

In 2018, the collaboration between NAV and specialist health services began to offer a new interdisciplinary program called *HelseIArbeid*, or *Health in work* (HIW) [22]. The program is one of the policy instruments in the latest Inclusive Work Agreement [17] and has elements of both IWM and “raskere tilbake,” a quick-access outpatient service intended to reduce sick leave by cutting wait times, which was in effect during 2007-2018. Thus, the HIW program has 2 main parts. The *individual measure* is an outpatient service where individuals can obtain quick access to an interdisciplinary assessment of common musculoskeletal, mental health complaints, or both, with a focus on how to better cope with these complaints to continue to engage in work and leisure activities. The second part is called the *company measure*, an interdisciplinary workplace intervention consisting of workplace processes regarding work environment as well as structured evidence-based health information about musculoskeletal and mental health complaints in general work contexts and related to the specific workplace [22]. Personnel from NWCs and specialist health services work in collaboration to deliver the intervention. This trial investigates the *company measure*, hereafter referred to as the HIW intervention. This trial is registered in ClinicalTrials.gov (NCT04000035).

The intersectoral and interdisciplinary collaboration between NAV and specialist health services, which is the core of the HIW intervention, is based on experiences from an earlier program called *iBedrift,* or *atWork*. The original *atWork* program started in some of Norway’s southern counties [8]. Its workplace interventions focused mainly on health information provided by health personnel. The *atWork* program further evolved in Troms County, where it was adapted into the interdisciplinary *atWork Troms* program, which was in effect during 2009-2017. The *atWork Troms* workplace interventions consisted of health information about musculoskeletal and common mental health complaints provided by health personnel combined with work processes outlined in the Inclusive Work Agreement guided and facilitated by NWC personnel. The recommendation for national implementation of the HIW intervention studied in this trial is closely related to the *atWork Troms* program [22].

### Rationale of the Trial

An evaluation of the Inclusive Work Agreement for the 2014-2018 period concluded that there was a lack of studies on workplace processes [15], including those presented in IWM interventions. Participants gave good feedback regarding the collaboration between health and NAV personnel in *atWork Troms* interventions, but no scientific evaluation of these interventions had been done. We know that certain parts of such interventions, such as providing health information at the workplace, can reduce sickness absence, increase self-rated work ability, and reduce physical and mental health complaints [8,23]. However, knowledge is still lacking about the effects and economic aspects of interventions integrating health information with workplace process assignments from NAV.

IWM interventions are customized according to the company’s needs and requests; thus, they can range from comprehensive interventions to simple ones that require little or no time or resources. In contrast, owing to their interdisciplinary nature and the required participation of all employees, HIW interventions are resource-demanding. It is unknown whether the costs of HIW interventions outweigh those related to sick leave and lost quality of life. Cost-effectiveness and cost-benefit analyses will provide an indication as to whether HIW is reasonable from a health and socioeconomic standpoint. Analyses of changes in outcomes related to health and the work environment will provide further information about the effect of HIW interventions.

Data on how the HIW intervention is perceived by both participants and intervention personnel will be gathered in the qualitative part of the overall mixed methods project, which is not described in this paper. The combination of qualitative and quantitative research methods will provide a more comprehensive picture of HIW interventions and may provide evidence of potential improvements to these interventions. The Norwegian Directorate of Health and NAV highlighted the need for knowledge about the effect of measures that combine the perspective of health and work [24] and encouraged quantitative and qualitative research to optimize the implementation and content of the *Health in work* program [22]. Therefore, this trial is an important contribution in the field and relevant for political decision makers, NAV, health services, and public and private workplaces.

### Specific Objectives

The main objective of this trial is to compare the effects of monodisciplinary IWM (treatment as usual) and interdisciplinary HIW interventions in terms of changes in overall sickness absence, health care use, health-related quality of life, and costs. The secondary objectives are to compare changes in individual sickness absence, psychosocial work environment, job and life satisfaction, health, and health anxiety at both the individual and group levels. Comparisons will be conducted both within and between trial arms.

## Methods

### Trial Design and Setting

This protocol presents a pragmatic, cluster-randomized, multicenter superiority trial with 2 parallel arms and a 1:1 allocation ratio. HIW and IWM interventions are to be carried out at workplaces throughout the county of Troms and Finnmark, Norway’s northernmost county, which covers an area of approximately 750,000 km^2^ and has a population of approximately 243,000 people. Workplaces in both urban and rural areas are included.

As both IWM and HIW interventions are aimed at all employees within a workplace, randomization must be conducted at the workplace level rather than at the level of individual employees.

### Eligibility and Exclusion Criteria for Clusters (Workplaces) and Individual Participants

To be eligible for inclusion, workplaces must have a minimum of 8 employees and have accessible data on sickness absence (both self- and physician-certified) for the 2 years before allocation. Individual participants must speak Norwegian, be aged 18-70 years, and be employed ≥20% in the participating workplaces to be included in the trial. Workplaces that are experiencing profound reorganization (ie, >20% change in workplace staff during the research period) are excluded.

### Interventions

#### HIW Intervention

Personnel from the NWCs and specialist health services work in collaboration to provide HIW interventions according to a standardized protocol. An initial meeting is held to define the workplace’s goals and plan the course of the intervention, followed by three 1.5-hour sessions over a 12-month period. During these sessions, health personnel present structured health information about musculoskeletal, pain, and mental disorders, and NAV personnel put this information in the context of work and the specific workplace. Between sessions, group-based workplace process exercises are carried out. During these exercises, challenges related to session content as well as topics around the work environment and work inclusion should be addressed.

In more detail, the first session aims to use health information on common neck and back complaints to increase workers’ ability to cope with them as individuals and collectively. It also presents tasks that foster reflection and dialogue on factors that promote the health and inclusion of workers with musculoskeletal complaints in the workplace. The second session aims to communicate health information regarding pain to increase workers’ ability to cope with it individually and collectively. The third session is similar to the first but with content related to common mental health complaints.

Both the sessions and the group exercises between the sessions should involve all workplace staff (management and employees). The group exercises should be active meetings that take place without the involvement of intervention personnel. Finally, at the end of the 3 sessions, an evaluation meeting is held to discuss possible further follow-up.

#### IWM Intervention (Treatment as Usual)

**Figure 1:**
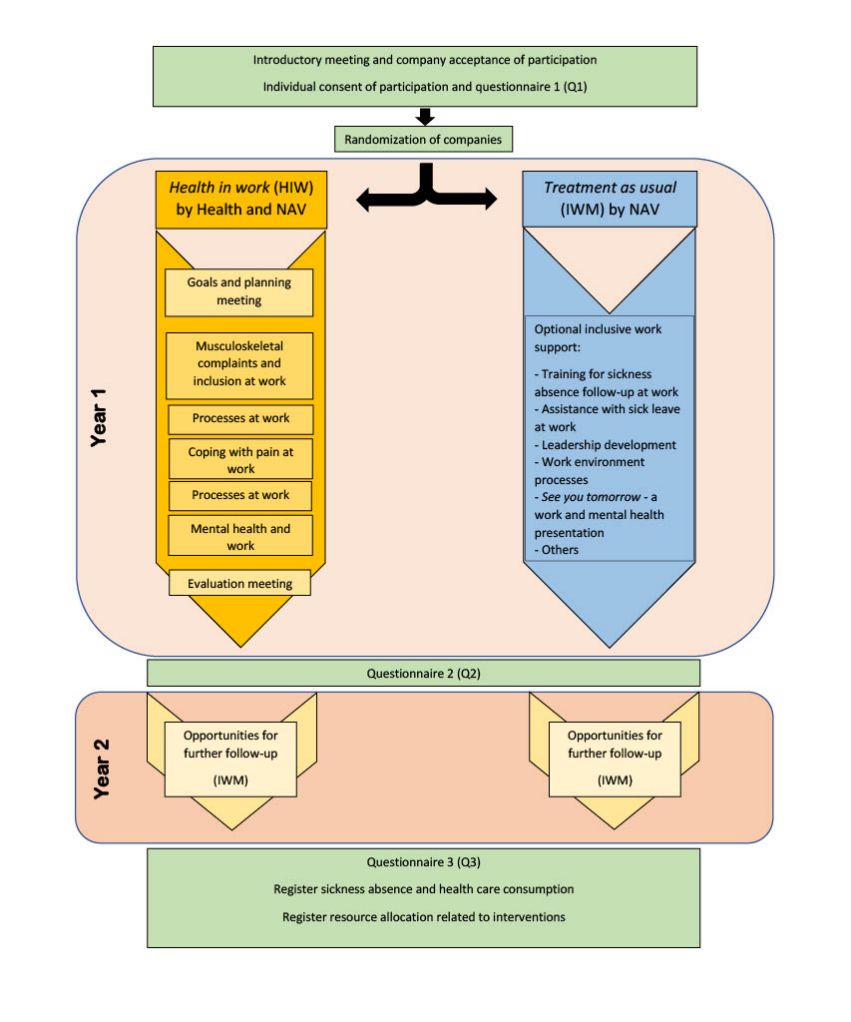
Schematic overview of activities in the *Health in work* (HIW) and *inclusive work measure* (treatment as usual) interventions. IWM: inclusive work measure; NAV: Norwegian Labour and Welfare Administration.

IWM consists of conventional welfare interventions given over a 12-month period by NWC personnel only. The interventions focus mainly on work inclusion and psychosocial work environment. There are several types of interventions available, some of which are presented in Figure 1. The choice of delivery is customized to the individual workplace; thus, treatment as usual varies according to the workplace’s demands. An evaluation meeting discussing possible further follow-up is optional.

A flowchart of intervention content and the study process is shown in Figure 1.

### Intervention Personnel

Health personnel comprise employees from specialist health services from the University Hospital of North Norway (UNN), Finnmark Hospital in Kirkenes, or the Rehabilitation Center Finnmark in Alta. They work in the field of rehabilitation medicine; for example, as physiotherapists, medical doctors, or occupational therapists. NAV personnel are from NWCs located at multiple sites in the county of Troms and Finnmark. They are experienced in Inclusive Work Agreement processes and have different backgrounds (eg, teaching, management, human resources, and nursing). Many have previously delivered *atWork Troms* interventions. NWC personnel deliver both HIW and IWM interventions according to their portfolio. All intervention personnel undergo in-house training according to protocol, including observation of experienced personnel, literature, and courses.

### Adherence to the Trial Protocol and Concomitant Activities

Participating NWC personnel, health personnel, and workplaces are to register and report on activity and time spent on either HIW or IWM each quarter. The research team sends reminders about these reports as a means of improving adherence.

Participating workplaces in both groups are allowed to implement other workplace measures (eg, measures from occupational health services or from NAV). The pragmatic design does not restrict the type or number of other workplace measures except for the HIW intervention, which is exclusively reserved for the HIW group. The content and extent of the applied measures have to be reported to the research group. Individual employees have no study-specific restrictions.

### Recruitment

The main role of NWCs is to support workplaces that wish to address the development and maintenance of a good work environment independent of their level of sickness absence. Workplaces can contact NWCs, but NWCs also reach out to workplaces. NWC personnel have good knowledge of and contact with workplaces in the region. Through this contact, workplaces from the county of Troms and Finnmark were recruited for the trial. They were informed about the ongoing trial and the fact that they may be allocated to either the HIW or IWM intervention in an introductory meeting with employers and union representatives. After initial slow recruitment, an additional meeting with detailed information solely on the trial and its implications given by one of the research group members (mainly CLT, occasionally NF or ACH) was added to increase participation.

### Timeline

**Figure 2:**
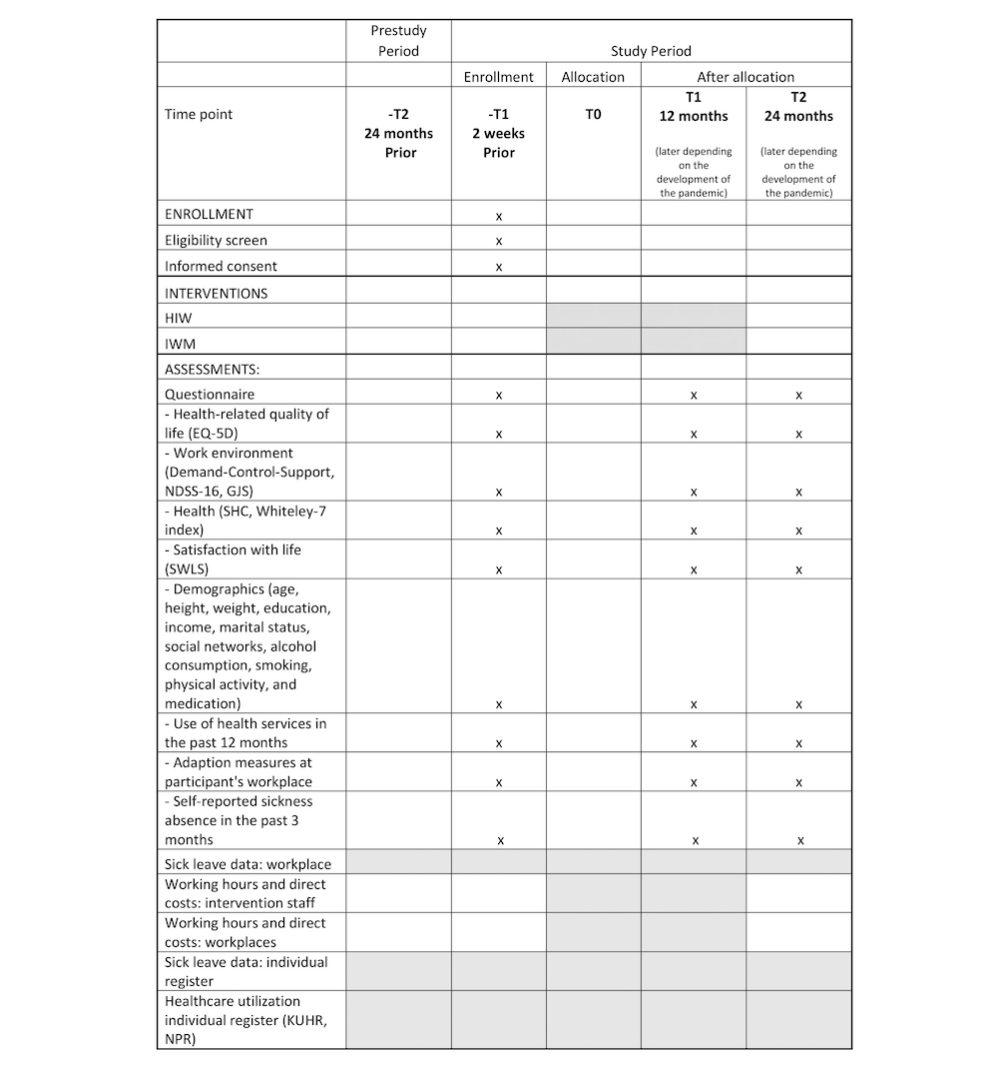
Timeline for enrollment, intervention, and assessment. GJS: Global Job Satisfaction; HIW: *Health in work*; IWM: inclusive work measure; KUHR: Norway Control and Payment of Health Reimbursement; NDSS–16: Nondirective and Directive Support Survey; NPR: Norwegian Patient Register; SHC: subjective health complaints; SWLS: Satisfaction With Life Scale.

Upon agreement to participate in the trial, all employees and management at the workplaces received the following by email from their employer: information about the trial, a PDF file with the individual informed consent form, and a link to questionnaire 1. Completed paper forms with individual informed consent were collected for 2 weeks, and then the random allocation was executed. Intervention periods were scheduled to be 12 months for both trial arms. Questionnaires 2 and 3 were to be completed 12 months and 24 months after allocation, respectively. Owing to the global pandemic, the trial was halted between mid-March 2020 and September 2020, and activities were reduced at several time points. Therefore, the intervention period and time points for questionnaires 2 and 3 were adjusted for workplaces included before April 2020 and in fall 2020, whereas workplaces included in 2021 were expected to follow the usual timeline. A flowchart of the study process is shown in Figure 1, and the timeline is shown in Figure 2.

### Assignment of Intervention

The randomization sequence was created using a computer-generated random number list [25] with a 1:1 allocation using random block sizes of 16 to 20. On the basis of this allocation, a sheet of paper was marked with either *HELSEIARBEID* (HIW) or *KTRIAARBEID* (IWM). The paper was then folded and placed in a sequentially numbered opaque envelope, and the envelope was sealed (allocation envelope). In this manner, a total of 104 allocation envelopes were produced. In November 2020, owing to an unexpectedly high number of retractions after initial recruitment but before inclusion in the trial, another 50 allocation envelopes were produced. Sequence generation and allocation concealment were performed independently by co–principal investigator (PI) ACH, who was not involved in the recruitment or allocation of workplaces. A list of the envelope numbers and the intervention they contained was locked away in a file cabinet at UNN, to which none of the personnel (research, NAV, or HIW personnel) has access. The workplaces were consecutively numbered as they agreed to participate in the trial during recruitment. Final inclusion was not completed until informed consent forms were received from the respective workplaces. The corresponding numbered allocation envelope was then opened by the PI, NF, together with a research assistant. The workplace was notified of the intervention to which it had been allocated, as was the supervisor of the relevant NWC. The workplace identifier was noted on the allocation envelope and locked away in a file cabinet. Blinding beyond allocation was not relevant for obvious reasons.

### Data Collection and Management

Trial questionnaires are electronic and administered by REDCap (Research Electronic Data Capture; Vanderbilt University) tools. Completed questionnaires are double-checked manually with informed consent forms to guarantee that only the data of those who have provided consent are used. Informed consent forms contain three sections to obtain separate consent for each of the following: use of questionnaire data, linkage to register data, and linkage to data from Tromsø 7, if applicable (Multimedia Appendix 1).

When answering questionnaire 1 at enrollment, participants registered their email address, to which links for later questionnaires will be mailed. Questionnaires 2 and 3 are completed at 12 and 24 months after allocation, respectively, or as appropriate owing to pandemic-related delays.

Questionnaires 1-3 all have the same questions on health-related quality of life, symptoms, work environment, and individual self-certified sickness absence. Validated questions were used when possible (see *Evaluation Outcomes* and Figure 2). Individual physician-certified sickness absence and health care use data are collected through register data for the period from 24 months before allocation to 24 months after allocation.

During the intervention period, workplaces report the time used for intervention activities on a quarterly basis. The sickness absence rate at the unit level is reported for the periods 24 months before allocation to allocation, the intervention period, and 12-24 months after allocation. Intervention staff registers the time spent on the preparation and execution of the intervention.

Workplaces that withdraw from the interventions before completing the intervention period are asked to continue to report their sickness absence data, and the individual participants are encouraged to answer all questionnaires, enabling intention-to-treat (ITT) analysis.

Data are stored on a secure research server at UNN with restricted access, which will also be used for data analysis. The personal national identification number will be used to collect register data according to consent. Data access is restricted to the project research group.

Study-specific personal identifiers will be used for all data storage, linkage, and analysis. The key list of identifiers is kept separate from the remaining data; access is restricted to PI NF and co-PI ACH.

### Ethics Approval

This research will be carried out in compliance with the Helsinki Declaration of 1975 as revised in 2000 [26]. Personal confidentiality is guaranteed. Written informed consent forms will be collected from each participant. The consent forms emphasize the right to withdraw from the trial at any time without explanation according to the consent form template created by the Norwegian Regional Committee for Medical and Health Research Ethics (REC). Information about the trial is given orally in an information meeting as well as in written form, with the opportunity to ask questions via telephone or email.

This trial protocol has been approved by the REC (ID 15680) and is registered at ClinicalTrials.gov (NCT04000035). Possible substantial protocol modifications must be approved by the steering committee and the REC and will be registered in ClinicalTrials.gov. The participant-level data set will not be available for public access owing to General Data Protection Regulations. Metadata and statistical codes beyond those reported in publications will be available upon reasonable request.

### Statistical Analysis

#### Power Calculation

The calculation of the sample size for the number of workplaces is pragmatic and based on Odeen et al [8]. ITT sickness absence will be collected at the unit level, and a 10% reduction from 6.6% (mean certified sickness absence in Troms) to 5.9% (SD 2.5%) will be considered significant in both a clinical and societal context. Odeen et al [8] demonstrated a statistically significant 11% difference in rate ratios at the unit level, with 42 and 48 units in the 2 arms. We consider actual units for inclusion as comparable and, therefore, plan to include 50 workplaces in each arm in line with the study by Johnsen [23] on workplace interventions in kindergartens.

At the individual level, the necessary sample size to detect a clinically significant difference within the arms was calculated according to the hypothetical use of the patient activation measure. A clinically significant difference in patient activation measure between the arms can be set at *D*=5 [27]. On the basis of this, and using a quantitative method where *f*(*a*=0.05, *b*=0.20)=10.5, we can calculate the necessary sample size for the trial arms: 182 (*n* = 2[*SD*/*D*]^2^ × *f*[*a*,*b*]) individuals in each arm if the SD is 17, strength (*b*) is 80%, and significance level (*a*) is 5%. Therefore, we need to include a total of 364 participants in the trial. To take into account the lack of independence between workers in clusters, this number should be corrected by *the variance inflation factor* 1 + (*n* − 1)*ρ*, where *n* is the average cluster size and *ρ* is the intracluster correlation coefficient for the particular outcome [28]. With the value of *n* being 4, the inflation factor equals 1.3 when *P* equals .10; thus, each trial arm should include 240 individuals. This indicates that, with a 50% response rate and a mean workplace size of 20 employees, we will have the power to perform gender-stratified analyses with 50 workplaces in each study arm.

#### Analysis of Data

Difference-in-difference analyses will be used to address any differences in sickness absence, health care refunds, and health-related quality of life between the trial arms at the unit level. Cost-effectiveness and cost-benefit analyses will compare the trial arms in terms of incremental costs based on direct and indirect costs related to interventions throughout the intervention period.

At the individual level, outcomes will be analyzed based on changes from enrollment. Moreover, 2-tailed *t* tests or Mann-Whitney *U* tests and chi-square tests will be used when appropriate. A generalized linear mixed effects Poisson model will be used to investigate possible differences in percentage of sick leave between the 2 trial arms. A multivariable, multilevel logistic, ordered logistic, and linear regression allowing for clustering at the unit level will be used according to the outcome measure (OM) analyzed. To consider changes owing to the pandemic-related temporary halt of the study, other statistical methods such as stratification will be relevant.

We will use 2-sided *P* values with *α*<.05 level of significance for all tests.

The analysis will be according to ITT regardless of protocol adherence.

Results will be reported in line with CONSORT (Consolidated Standards of Reporting Trials) guidelines.

### Evaluation Outcomes

The *Health in work* program and the Inclusive Work Agreement have several complex, nongraded aims, which focus on improved work environment, sustainable job participation, and prevention and reduction of sickness absence. The *Health in work* program especially pinpoints work participation as an aspect that contributes to better health. However, it is not known to what extent the systematic intersectoral collaboration between NAV and specialist health services in HIW interventions is an effective use of resources. Therefore, it is impossible to define a single primary OM for this trial and, thus, the four primary OMs are as follows: (1) change in overall sickness absence rates (self- and physician-certified) at the workplace (unit level) in percentage of planned workdays for the period 24 months before allocation compared with the period 12-24 months after allocation (OM 1); (2) change in health care use, assessed using data from the National Register of Control and Health Service Refunds (The Norwegian Directorate of Health) and the Norwegian Patient Register (The Norwegian Directorate of Health), for the period 24 months before allocation compared with the period 12-24 months after allocation (OM 2); (3) change in health-related quality of life, measured using the EQ-5D-5L and EQ VAS for the period from enrollment to 24 months after allocation (OM 3); for OMs 1-3, changes within and between trial arms will be analyzed); and (4) health economic analyses comprising cost-effectiveness analyses based on OM 3 and cost-benefit analyses based on OMs 1 and 2 comparing the trial arms in terms of incremental costs based on direct and indirect costs related to the interventions throughout the intervention period (OM 4).

Secondary OMs focus on health complaints, anxiety, and different aspects of work and work environment and comprise the following: change in physician-certified mean sickness absence rates (individual level) based on data from the Norwegian National Register of Sickness Absence (NAV sykefraværsregister) for the period 24 months before allocation compared with the period 12-24 months after allocation (OM 5); change in self-certified sickness absence rates (individual level; OM 6); change in psychosocial work environment, assessed using the Demand–Control–Support Questionnaire score (OM 7); change in social support from colleagues, assessed using the Nondirective and Directive Support Survey score (OM 8); change in job satisfaction, assessed using the Global Job Satisfaction score (OM 9); change in subjective health complaints, assessed using the subjective health complaints score (OM 10); change in health anxiety, assessed using the Whiteley index score (OM 11); and change in satisfaction with life, assessed using the Satisfaction With Life Scale score (OM 12).

OMs 6-12 are based on data from the trial questionnaires. Changes within and between trial arms will be analyzed for the following periods: enrollment to 12 months after allocation and enrollment to 24 months after allocation for each OM and 12 to 24 months when relevant.

For in-depth details regarding OMs, see ClinicalTrials.gov.

The trial questionnaire collects information on background variables (ie, type of employment, height, weight, education, income, smoking habits, alcohol consumption, and physical activity) as well as self-reported sickness absence and health care use. Several sections of the trial questionnaire are the same as those in the seventh and latest survey of the Tromsø Study (Tromsø 7), which took place in 2015-2016. For trial participants who also participated in Tromsø 7 and provided explicit consent, trial data will be linked to Tromsø 7 data to obtain an impression of natural intraindividual variation over time.

For all analyses, the possible impact of the COVID-19 pandemic will be addressed.

### Patient and Public Involvement

Patients and the public have been involved in the design of and recruitment for the study by representatives of employer and employee organizations both in the preparation phase and in the steering committee. They will be further involved in dissemination activities to ensure that information is clearly given and easy to understand for our different audiences.

### Steering Committee and Reference Group

The overarching mixed methods project has a steering committee with members from a Labour Union (Landsorganisasjonen i Norge [LO]) and an employer union (The Confederation of Norwegian Enterprises [NHO]) as well as funders and sponsors NAV research and development fund, UNN, UiT The Arctic University of Norway, and NWCs). The committee is chaired by NAV Troms and Finnmark, which is guiding the practical execution of the interventions. A reference group comprising representatives from the research group, NAV research and development fund, and intervention participants as well as researchers from *Stiftelsen for industriell og teknisk forskning* (SINTEF) and the University of South-Eastern Norway advises on scientific questions.

## Results

Inclusion began in June 2019 and was completed in July 2021, resulting in 97 included workplaces and 1383 individual consents. Recruitment of workplaces was difficult. Of the 146 recruited workplaces that had initially agreed to participate, only 97 (66.4%) finally consented to inclusion, whereof 46% (45/97) were allocated to HIW and 54% (52/97) were allocated to IWM. Reasons given for retraction were both pandemic-related and organizational challenges that would have made it difficult for the workplaces to satisfy the study requirements.

Questionnaire 1 was answered by 962 participants, yielding a response rate of 69.6% (962/1383). Although the time point for questionnaire 2 has been reached for only a part of the participants, none have received questionnaire 3 as of February 20, 2022. Data collection will be completed with the last questionnaires to be sent out in July 2023. Results on intervention-related difference-in-difference regarding self-rated health measures, sickness absence, and health care use are expected to be published from autumn 2023 onward.

## Discussion

### Preliminary Principal Findings

This trial will examine different aspects of workplace interventions and specifically compare the new interdisciplinary HIW program with usual monodisciplinary IWM in terms of health care use, health-related quality of life, and costs. As secondary objectives, it will also compare changes in individual sickness absence, psychosocial work environment, job and life satisfaction, health, and health anxiety at both the individual and group levels.

Although the number of finally enrolled workplaces was slightly below our desired target of 100 workplaces, individual participant numbers were well above the anticipated goal of 1000 enrolled participants. Despite computer-generated randomization, the distribution to the 2 intervention groups was slightly skewed toward the IWM group but not significantly different.

To date, the response rate for our questionnaire can be considered good, being 69.6% (962/1383), but response rates for questionnaires 2 and 3 will likely be lower. Incentives have proven to increase participation rate in follow-up questionnaires in randomized controlled trials [29]. We are already applying incentives in the form of gift certificates for 750 Norwegian kroners (US $84.31), which are randomly drawn from every 50 questionnaires received since the start of the study. Information about this was provided in the original email before questionnaire 1 and is included in emails for follow-up questionnaires 2 and 3.

### Strengths and Limitations

Our trial is the first large-scale scientific evaluation of the *Health in work* program, which has thus far not been scientifically evaluated. The pragmatic design will reflect the effects that can be achieved by the 2 interventions in real life. The combined use of questionnaires and register data is a strength that could ameliorate the negative impact that a possible low response rate to follow-up questionnaires would have on representativeness.

A major strength is the trial design, with random allocation to one of the 2 intervention groups. As allocation was conducted by persons not involved in the production of allocation envelopes and only after individual consent forms were received, we have ameliorated the chances of selection bias (ie, the selective enrollment into the trial based on likelihood of the next treatment allocation). In addition, during the recruitment period, NWCs offered HIW only to workplaces participating in the trial (with a 50% chance of receiving HIW or IWM). IWM support was administered independent of trial participation. However, this could also have introduced a bias in that workplaces that wanted to receive HIW but were allocated to IWM will be less motivated for IWM activities or answering questionnaires. Attention will have to be given to possible differences in response rates of follow-up questionnaires in the HIW and IWM groups.

A potential limitation is that there might occur cross-contamination between trial arms, between nearby workplaces, and because of personnel serving both HIW and IWM interventions. In addition, generalizability could be influenced by regional workplace culture and characteristics, such as the experience of executing staff from NAV and specialist health care services.

### Dissemination Plan

Trial results will be disseminated to participants, researchers, health personnel, authorities, and others interested through scientific conferences, publications, reports, and public dissemination measures. There are no publication restrictions, and the results will be disseminated regardless of the magnitude or direction of the effect. Authorship eligibility is determined according to the International Committee of Medical Journal Editors criteria for manuscripts submitted for publication. There is no intended use of professional writers.

Data presentation will be performed in a way that ensures confidentiality for individual or workplace-specific data.

## Appendix

Multimedia Appendix 1 Administrative information about the trial, including the World Health Organization trial registration data set and the trial’s informed consent form in original language (Norwegian).

## Abbreviations

CONSORT: Consolidated Standards of Reporting Trials
HIW: Health in work
ITT: intention-to-treat
IWM: inclusive work measure
LO: Landsorganisasjonen i Norge
NAV: Norwegian Labour and Welfare Administration
NHO: Confederation of Norwegian Enterprises
NWC: Norwegian Labour and Welfare Administration work center
OM: outcome measure
PI: principal investigator
REC: Regional Committee for Medical and Health Research Ethics
REDCap: Research Electronic Data Capture
SINTEF: Stiftelsen for industriell og teknisk forskning
UNN: University Hospital of North Norway
