# Supplementary material for: Comparing the New Interdisciplinary Health in Work Intervention With Conventional Monodisciplinary Welfare Interventions at Norwegian Workplaces: Protocol for a Pragmatic Cluster Randomized Trial
Source: JMIR Res Protoc. 2022 Apr 7;11(4):e36166. doi: 10.2196/36166 (PMC9030981; doi:10.2196/36166)
Supplement: Multimedia Appendix 1 [file resprot_v11i4e36166_app1.pdf]

Administrative information
The trial is registered at clinicaltrials.gov (NCT04000035)

For items from the World Health Organization Trial Registration Data Set, see table 1

Protocol version: Version 3 due to pandemic-related changes in content and timeline/ timepoint of questionnaire.

Funding sources: The project is funded by the Norwegian Labour and Welfare Administration's research and development fund (NAV FoU-2018 announcement), contributions from the Helse Nord Health Trust and UiT The Arctic university of Norway. These funds cover material and personnel costs related to research. Studied interventions are offered as ordinary health and welfare-services and are thus no issue of project funding.

Roles and responsibilities – sponsor contact information
Norwegian Labour and Welfare Administration Troms and Finnmark, Postboks 6070 Langnes, 9290 TROMSØ

Roles and responsibilities – sponsor and funder
NAV work center is responsible for enrollment to the study and to co-organize interventions with health personnel at UNN, but has no influence on randomisation, collection, management, analysis or interpretation of data, writing of the report, the decision to submit the report for publication or ultimate authority over any of these activities.

Table 1 WHO Trial Registration Data Set (Version 1.3.1)

**Primary Registry and Trial Identifying Number**	clinicaltrials.gov https://clinicaltrials.gov/ct2/show/NCT04000035
**Date of Registration in Primary Registry**	June 11th, 2019
**Secondary Identifying Numbers**	REK Nord ID 15680
**Source(s) of Monetary or Material Support**	Norwegian Labour and Welfare Administration's research and development fund (NAV FoU-midler), contributions from the Helse Nord Health Trust and UiT The Arctic University of Norway
**Primary Sponsor**	University Hospital of North Norway UNN
**Secondary Sponsor(s)**	The Labour and Welfare administration of Troms and Finnmark county, and UiT The Artic University of Norway
**Contact for Public Queries**	University Hospital of North Norway, Tel : +47 77644342; hiaforsk@unn.no

**Contact for Scientific Queries**	Principal investigator:<br>Nils Fleten, MD/PhD, senior consultant at NAV Troms and Finnmark, and associated professor UiT The Artic University of Norway;<br>nils.fleten@uit.no<br>Tlf +47 92256168<br>Delegated responsibility of RCT:<br>Anje C Höper, MD/PhD<br>Dpt of Occupational and Environmental medicine<br>University Hospital North Norway<br>Postboks 16<br>9038 Tromsø<br>Tel : +47 77644342<br>hiaforsk@unn.no
**Public Title**	*Health in work*- a path to improved health and work environment?
**Scientific Title**	Comparing the new interdisciplinary Health in work intervention to conventional monodisciplinary welfare interventions at Norwegian workplaces: Protocol for a pragmatic cluster-randomized trial
**Countries of Recruitment**	Norway
**Health Condition(s) or Problem(s) Studied**	Common health complaints including mild mental disorders in the light of work inclusion
**Intervention(s)**	Health in work (HIW): Interdisciplinary workplace intervention. Health- and welfare personnel (intervention staff) provide information on how to handle common health disorders at the workplace, according to study protocol. In addition, workplace staff is to work with work environment in groups guided by intervention staff.<br>Treatment as usual (IWM): Monodisciplinary workplace intervention. Contents vary depending on needs and desires of workplace. Can include protocolised information interventions and self-work with work environment. Intervention staff consists of welfare personnel only.
**Key Inclusion and Exclusion Criteria**	**Healthy human volunteers**<br>**For workplace units:** Inclusion: minimum 8 employees, accessible data on sickness absence in the unit for the past two years prior to inclusion. Exclusion criteria are ongoing profound re-organisation at the workplace or

	workplace unit (>20% change of staff within workplace unit).<br><br>**For individuals:** Inclusion: Norwegian-speaking, age 18-70 of all sexes, employed > 20% in the participating workplaces/workplace units.
**Study Type**	Interventional<br>Allocation: cluster-randomized<br>Intervention model: two-arm parallel assignment<br>Primary purpose: prevention
**Date of First Enrollment**	June 14th, 2019
**Sample Size**	100 workplaces and minimum 1000 individual respondents answering questionnaires.
**Recruitment Status**	Recruiting completed. 97 workplace units and 1383 individual respondents included.
**Primary Outcome(s)**	Change in sickness absence, healthcare utilization, Health-related quality of life; health-economic analyses
**Key Secondary Outcomes**	Change in health complaints, health anxiety, work environment and satisfaction of life
**Ethics Review**	Norwegian Regional Committee for medical and health research ethics North ID 15680 (approved 21.12.2018; rek-nord@asp.uit.no
**Completion date**	Completion of data will be 2 years from last enrolment (July 2023)
**IPD sharing statement**	No

FORESPØRSEL OM DELTAKELSE I FORSKNINGSPROSJEKTET

HelseIArbeid - et tiltak for økt mestring og arbeidsdeltagelse?

«En analyse av virkning, kostnader og effekt av forebyggende kunnskapsformidling på arbeidsplassen.»

Dette er et spørsmål til deg om å delta i et forskningsprosjekt for å teste effekten av arbeidsplasstiltaket HelseIArbeid. Du mottar dette spørreskjemaet fordi din arbeidsplass/bedrift har sagt «ja» til å delta i dette prosjektet.

HVA INNEBÆRER PROSJEKTET?

HelseIArbeid er en nasjonal satsing på samarbeid mellom NAV, Helsetjenesten og Arbeidslivet for å øke inkludering, mestring og arbeidsdeltagelse. I HelseIArbeid tiltaket formidles oppdatert kunnskap om muskel/skjelett- og psykiske helseproblemer til alle ansatte og ledere. I tillegg skal man jobbe med arbeidsmiljøet og med å anvende denne kunnskapen på arbeidsplassene.

I dette forskningsprosjektet skal HelseIArbeid sammenlignes med vanlige inkluderende arbeidsliv (IA-) oppfølgingstiltak fra NAV arbeidslivssenter. Arbeidsplasser som takker «ja» til å bli med i dette forskingsprosjektet vil etter loddtrekking enten få tilbud i tråd med HelseIArbeid tiltaket eller vanlig IA-oppfølging.

I prosjektet vil vi innhente og registrere opplysninger om deg ved hjelp av spørreskjema og registerkobling. Spørreskjemaer blir sendt ut når arbeidsplassen din starter i forskningsprosjektet og deretter to ganger til i løpet av de følgende to år. Besvarelsen tar ca. 20-30 min. Vi ønsker å se på om det skjer endringer i ansatte sin vurdering av eget arbeidsmiljø, mestring av arbeid og helseplager, samt sykefravær og bruk av helsetjenester.

For å se om det er endringer i sykefravær og helsetjeneste-forbruk, ønsker vi å koble opp mot noen registre (NAV sykefraværsregister, Kontroll og utbetaling av helserefusjoner – (KUHR-)database og Norsk pasientregister – NPR). Her ønsker vi å se på perioden to år før til to år etter oppstart av tiltaket. Denne informasjonen vil også brukes for å sammenligne kostnader og nytte ved de to tiltak.

For personer som har deltatt i Tromsøundersøkelsen 2015-16 (Tromsø 7), vil vi sammenstille data fra det aktuelle spørreskjemaet med data fra Tromsøundersøkelsen. Dette for å kunne belyse utvikling i egenvurdert helse og helsebekymring.

MULIGE FORDELER OG ULEMPER

Som deltaker i prosjektet kan du være med på å gi ny kunnskap om nytteverdi av forskjellige arbeidsplasstiltak. Hvis din bedrift er med på HelseIArbeid tilbud, vil du motta et nytt tilbud på arbeidsplassen, blant annet foredrag om alminnelige helseplager.

I kontrollgruppen med vanlig IA oppfølging fra NAV arbeidslivssenter vil deltagelse kunne bidra til økt fokus på arbeidsmiljøspørsmål i bedriften.

Hvis du samtykker, må du regne med å bruke 20-30 minutt på å fylle ut spørreskjemaet. Dette skal gjøres tilsammen tre ganger i løpet av to år.

FRIVILLIG DELTAKELSE OG MULIGHET FOR Å TREKKE SITT SAMTYKKE

Det er frivillig å delta i prosjektet. Dersom du ønsker å delta, undertegner du samtykkeerklæringen på siste side. Du kan når som helt og uten å oppgi noen grunn trekke ditt samtykke. Dette vil ikke få konsekvenser for din behandling i helsevesenet eller NAV. Dersom du trekker deg fra prosjektet, kan du kreve å få slettet innsamlede opplysninger, med mindre opplysningene allerede er inngått i analyse eller brukt i vitenskapelige publikasjoner. Dersom du senere ønsker å trekke deg kan du kontakte Christoffer Terjesen på telefon 77626930 eller Anje C Höper på telefon 77644342 eller skrive epost til hiaforsk@unn.no.

HVA SKJER MED OPPLYSNINGENE OM DEG?

Opplysningene som registreres om deg skal kun brukes slik som beskrevet i hensikten med prosjektet. Du har rett til innsyn i hvilke opplysninger som er registrert om deg og rett til å få korrigert eventuelle feil i de opplysningene som er registrert. Du har også rett til å få innsyn i sikkerhetstiltakene ved behandling av opplysningene.

Alle opplysningene vil bli behandlet uten navn og fødselsnummer eller andre direkte gjenkjennende opplysninger. En kode knytter deg til dine opplysninger gjennom en navne- og epostliste. Tilsvarende lages en liste som knytter koden til ditt personnummer når du samtykker til kobling mot NAV, KUHR-register, NPR og/eller Tromsøundersøkelsen 2015-16. Det er kun prosjektlederne Anje Christina Höper og Nils Fleten som har tilgang til de to listene.

Opplysningene om deg vil bli anonymisert eller slettet senest fem år etter prosjektslutt.

FORSIKRING

Deltakelse vil inngå i ordinært arbeidsmiljøarbeid og vil dermed være dekket av arbeidsgivers forsikringsordninger.

GODKJENNING

Regional komité for medisinsk og helsefaglig forskningsetikk har vurdert prosjektet, og har gitt forhåndsgodkjenning REK Nord 2018/2262.

Etter ny personopplysningslov har behandlingsansvarlig Universitetssykehuset Nord-Norge og prosjektleder Anje Christina Höper et selvstendig ansvar for å sikre at behandlingen av dine opplysninger har et lovlig grunnlag. Dette prosjektet har rettslig grunnlag i EUs personvernforordning artikkel 6a og artikkel 9 nr. 2 og ditt samtykke.

Du har rett til å klage på behandlingen av dine opplysninger til Datatilsynet.

KONTAKTOPPLYSNINGER

Dersom du har spørsmål til prosjektet kan du ta kontakt med:

- Christoffer Terjesen på telefon 77626930 eller
- Anje C Höper på telefon 77644342
- eller skrive epost til hiaforsk@unn.no

Personvernombud ved institusjonen er personvernombudet@unn.no.

JEG SAMTYKKER TIL Å DELTA I PROSJEKTET OG TIL AT MINE PERSONOPPLYSNINGER BRUKES SLIK DET ER BESKREVET

____

Sted og dato Deltakers signatur

____

Deltakers navn med trykte bokstaver

____

Deltagers epostadresse

JEG SAMTYKKER TIL KOBLING MED FØLGENDE REGISTER (KRYSS AV):

	Ja	Nei
Jeg har deltatt og samtykker til **kobling mot Tromsøundersøkelsen** 2015-16		
Jeg samtykker til **uthenting av** legemeldt **sykefravær** fra NAV sine register for sykefravær		
Jeg samtykker til **uthenting av helsetjenesteforbruk** fra KUHR databasen og Norsk pasientregister		

____

Sted og dato Deltakers signatur

____

Deltakers personnummer
